# Nutrition-Related Knowledge, Attitudes, Practices, and Anemia Status of Lactating Mothers in Bukavu, Democratic Republic of the Congo—A Cross-Sectional Analysis

**DOI:** 10.3390/nu16060870

**Published:** 2024-03-17

**Authors:** Damaris Elisabeth Beitze, Céline Kavira Malengera, Theophile Barhwamire Kabesha, Veronika Scherbaum

**Affiliations:** 1 Department of Food Biofunctionality, Institute of Nutritional Sciences, University of Hohenheim, 70599 Stuttgart, Germany; veronika.scherbaum@uni-hohenheim.de; 2 School of Medicine and Public Health, Université Evangélique en Afrique, Bukavu B.P. 3323, Democratic Republic of the Congo; kavira.celine@gmail.com (C.K.M.); theo_kabesha@yahoo.fr (T.B.K.); 3 Département de Nutrition, Centre de Recherche en Sciences Naturelles/Lwiro, Bukavu, Democratic Republic of the Congo; 4 Faculty of Medicine, Official University of Bukavu, Bukavu, Democratic Republic of the Congo

**Keywords:** nutritional knowledge, nutritional attitudes, dietary diversity, anemia, food choice, lactating mothers, infants, DR Congo

## Abstract

Maternal nutrition is impacted by personal and environmental factors including dietary intake, knowledge, food availability, and affordability. This cross-sectional analysis aimed to evaluate nutrition-related knowledge, attitudes, practices, and associations with hemoglobin concentration among lactating mothers in the Bukavu region, Democratic Republic of the Congo. In 444 lactating mothers, nutrition-related knowledge and practice were assessed by questionnaires and translated into knowledge and practice scores ranging from 0 to 1, attitudes and drivers of food choice were assessed, the Dietary Diversity Score (DDS) was assessed with 24 h dietary recalls in a potential range from 0 to 10, and hemoglobin (Hb) was measured in mothers and their infants. Anemia prevalence was 28.2% among mothers and 74.3% among infants aged 3–8 months. Nutritional knowledge and practice were limited (the median total knowledge score was 0.39, median DDS was 3.0). While there were slight positive correlations between knowledge and maternal Hb, DDS did not significantly correlate with either knowledge or Hb. Although half of the mothers stated a perception about their own susceptibility to anemia or vitamin A deficiency (56.4%, 47.4%), less than half of those could justify their estimation (40.9%, 44.2%). Taste (68.1%), appearance (42.5%), availability (29.0%), and health effects (25.6%) were important drivers of food choice. In conclusion, interventions on the different influencing factors including nutrition education strategies are needed.

## 1. Introduction

Dietary behavior is impacted by various factors that include personal taste preferences, previous experiences, attitudes, and knowledge, but also environmental factors such as food availability, cultural practices, and price [1,2]. An understanding of the drivers of food choice, existing knowledge, and beliefs is required for targeted combating of malnutrition. The FAO recommends assessing nutrition-related knowledge, attitudes, and practices in order to identify gaps within them and plan nutrition education interventions [2].

A nutritionally susceptible period is the 1000 day-window of opportunity, covering pregnancy and the first two years of a child’s life [3,4]. Lactating mothers play an important role by providing breast milk and complementary food to their children. In terms of nutrient concentrations, breast milk quality is compromised by maternal undernutrition for some micronutrients, while for others the nutrient concentrations remain stable, but maternal reserves may be depleted [5]. Studies found a higher anemia risk for lactating compared to non-lactating mothers [6,7].

The Democratic Republic of the Congo (DRC) faces high levels of underweight and anemia among women aged 15–49 years (14.4%, 38.4%) and children aged 6–59 months (22.6%, 59.8%), according to the latest Demographic Health Survey (DHS) in 2013–2014 [8]. In South Kivu, studies reported an anemia prevalence of 16.5–17.6% in pregnant and non-pregnant women of reproductive age (DHS: 22.7%) and 47.8–58.6% in children aged 6–23 months (DHS: 35.7% in children 6–59 months) [8,9,10,11]. Also, in South Kivu, Moumin et al. [12] found a moderate prevalence of inadequate vitamin A (18%) and iron intake (33%) among women of reproductive age, and moderate to high inadequacy of further nutrients such as folate or zinc. Despite this evidence on poor diet in the region, to our knowledge, evidence on the determinants of dietary practices such as nutritional knowledge and attitudes, and their potential relationship with anemia status is scarce, especially for lactating women.

This study aimed to (a) explore the status of nutritional knowledge, attitudes, and practices among lactating mothers around Bukavu, DRC; (b) evaluate interrelationships between them and with maternal factors; and (c) evaluate associations with hemoglobin concentration.

## 2. Materials and Methods

This study is reported according to the STROBE recommendations [13].

### 2.1. Study Design and Setting

**Figure 1:**
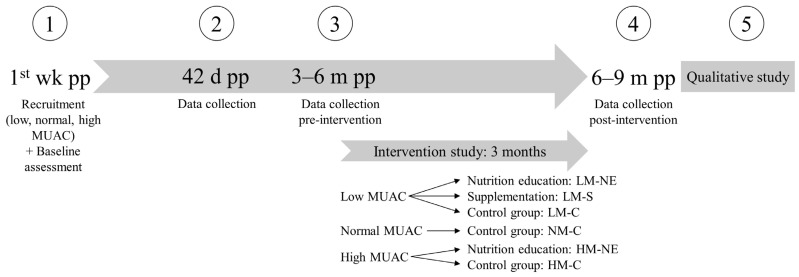
Study design. d: days, m: months, MUAC: mid-upper arm circumference, pp: postpartum, wk: week. 1–5: assessment points.

This multicenter cross-sectional evaluation is part of a larger project conducted among lactating mothers and their infants in semi-urban and rural areas of Bukavu, Democratic Republic of the Congo between December 2017 and June 2019. A cross-sectional follow-up study was performed (Figure 1). Mother–infant pairs were recruited in the first week postpartum in two semi-urban hospitals and one rural hospital and followed for 6–9 months with four assessment points, followed by a qualitative survey with a subgroup. Between the third and fourth assessment, an intervention study was implemented, whose results will be published elsewhere. The overall study aimed to evaluate and improve the health and nutritional status of mothers and infants. Details on the study setting have been previously published [14].

At the baseline, socio-demographic parameters were assessed [14]. At the third and fourth assessment (pre- and post-intervention), maternal mid-upper arm circumference (MUAC) and hemoglobin (Hb) concentration of mother and child were measured. A 24 h dietary recall and a pre-tested structured questionnaire regarding dietary practices, infant feeding practices, and nutritional knowledge and behavior of the mother were conducted. Data collection was performed by trained local health personnel in Swahili after developing the material in English, translating it to French, and finally to Swahili. In case of severe underweight and/or anemia, mothers and infants were referred to a hospital for treatment but remained in the study.

This work presents results for the third assessment at 3–6 months postpartum as a cross-sectional analysis. Data were collected prior to the intervention in health centers belonging to the study hospitals of recruitment. The period of data collection covered dry and rainy seasons.

### 2.2. Study Participants

Mothers aged 18–45 years delivering a healthy, full-term, single newborn in one of three study hospitals were enrolled with their newborn on condition of written informed consent. Mother–infant pairs were not eligible to participate if mothers were severely underweight, suffered from an immunodeficiency disease in the last stage, experienced complications during pregnancy, or were not living in the catchment health areas/not visiting one of the study health centers for follow-up appointments. Further details are presented elsewhere [14].

Sample size calculation and recruitment were based on the intervention study’s requirement of three groups of mothers with low MUAC (≥21 and <25 cm), one group with normal MUAC (≥25 and <28 cm), and two groups with high MUAC (≥28 cm). Accordingly, each eligible mother with low or high MUAC and every third mother with normal MUAC was recruited [14].

### 2.3. Outcomes

Maternal nutritional knowledge, attitudes, practices, dietary intake, MUAC, and maternal and infantile Hb were analyzed in this study. Maternal age and educational level were assessed at baseline [14] and included into analyses as they were previously reported to be associated with nutritional knowledge, dietary intake, or anemia [6,15,16,17,18].

#### 2.3.1. Nutritional Knowledge, Attitudes, and Practice

A structured questionnaire was developed for this study, following the FAO guidelines for assessing nutrition-related knowledge, attitudes, and practices [2], and pre-tested in the study area. To evaluate the impact of nutrition education in the intervention study, open-ended knowledge and practice questions covered topics taught during the intervention: knowledge on the three food group model, malnutrition, iron and anemia, vitamin A and vitamin A deficiency, and practices regarding iron (see Table 1). Answers on knowledge were classified as correct/wrong. Classification of answers regarding causes and prevention of malnutrition, anemia, and vitamin A deficiency was conducted according to the UNICEF Conceptual Framework on Maternal and Child nutrition [19], the UNICEF conceptual framework of the determinants of child undernutrition [20], the conceptual model of the determinants of anemia [21], and the conceptual framework of causes of vitamin A deficiency [22]. All immediate, underlying, and enabling/basic determinants were classified as correct. Nutritional practices were rated as beneficial/non-beneficial. Missing answers, questions that were not applicable, and questions that were not answered (for knowledge and practice) were excluded as missing values.

Knowledge and practice scores (K- and P-Scores) were developed for each topic (Table 1). A question was rated with 1 if at least one correct answer was given, and 0 if no correct answer was given (including do not know and wrong answers). For practices, beneficial practice was rated with 1 and non-beneficial with 0. Scores were calculated per knowledge/practice topic as the proportion of questions correctly answered out of all questions answered, ranging from 0 to 1. If the total number of questions answered was below 75% of all respective questions, no score was calculated. An overall knowledge score including all 19 questions was calculated (K-Score *total*).

Evaluation of attitudes equally covered the topics of malnutrition, iron and anemia, and vitamin A and vitamin A deficiency. Perceived susceptibility, severity, benefits, barriers, and self-efficacy towards conditions of malnutrition and preparation of nutrient-rich foods were assessed. Given justifications for their perceptions were categorized inductively. Mothers were further asked to report their reasons for dietary choices.

#### 2.3.2. Dietary Intake: 24 h Dietary Recalls

The 5-step multiple pass method [23] of the United States Department of Agriculture (USDA) was followed in conducting 24 h dietary recalls (24hDRs). Portion size estimation was facilitated by the use of household measures, pictures of local food portions, and food prices. Portion sizes of side ingredients in shared dishes were estimated by dividing the total amount of the ingredient used in preparation by the total number of persons consuming the meal. The 24hDRs’ dietary data were evaluated with EBISpro for Windows 2016 with the United States Department of Agriculture (USDA) database (SR 28). It was complemented by African databases for local foods [24,25,26]. Exceptionally, foods not contained in any of the databases were replaced by similar ones. In case of missing portion size estimations, e.g., if the dish had not been prepared by the mother herself, standard portions were applied. Supplement intake was not recorded.

Mothers were asked if they reported a normal day or a special occasion. Analysis considered normal days (*n* = 403) and special occasions such as visits to neighbors, fasting during part of the day, or unusual eating behavior at funerals as part of usual life (*n* = 8). Mothers who did not consume any food were excluded from analysis (*n* = 7).

Food groups contributing to the Dietary Diversity Score (DDS) were assigned to the reported foods according to the FAO [27] as follows: starchy staples; pulses; nuts and seeds; milk and milk products; meat, poultry, fish; eggs; dark green leafy vegetables; other vitamin A-rich fruits and vegetables; other vegetables; and other fruits. Foods not belonging to these groups were classified in the following groups: oils and fats; unhealthy foods (fried and salty foods, sweet foods, sweet beverages); alcoholic beverages; condiments and seasonings; other foods and beverages. Any food group was considered as consumed when it had an intake of at least 15 g over the whole day. Consumption of sub-groups was considered at lower amounts in case of an intake of at least 15 g of their main food groups. Anything consumed with less than 15 g was classified as a condiment or seasoning. The DDS was calculated as the sum of consumed food groups. Minimum Dietary Diversity for Women (MDD-W) was defined as consumption of at least 5 out of the 10 food groups [27].

#### 2.3.3. Anthropometrics and Hemoglobin

MUAC of the mother was measured with a non-stretchable measuring tape (seca 212; seca GmbH & Co. KG, Hamburg, Germany) to the nearest millimeter. Measurements were performed in repetition and repeated in case of a difference of >0.2 cm. Mean of measurements was calculated. Hemoglobin concentration was measured on site using the HemoCue Hb 201+ system (HemoCue AB, Ängelholm, Sweden) using finger (mothers) or heel pricks (infants).

Hb concentration was adjusted to altitude by −0.5 g/dL, but not to African ethnicity as there is no final decision about ethnic-specific cut-offs [28]. For non-pregnant mothers, mild, moderate, and severe anemia were defined as Hb = 11.0–11.9 g/dL, Hb = 8.0–10.9 g/dL, and Hb < 8.0 g/dL, respectively. For infants ≥ 6 months of age, mild, moderate, and severe anemia were defined as Hb = 10.0–10.9 g/dL, Hb = 7.0–9.9 g/dL, and Hb < 7.0 g/dL, respectively [28]. Because there are no official cut-offs set for children under 6 months of age, the same thresholds as for older children were used, in line with Safiri et al. [29]. Potential causes of anemia such as nutrient deficiencies, infections, or genetic Hb disorders were not assessed in this study.

### 2.4. Sample Size and Statistics

The required sample size was calculated to assess the difference in maternal hemoglobin (Hb) concentration between the intervention groups at post-intervention according to Allen, 2011 [30]. Based on the assumptions of a Hb difference of Δ = 0.37 g/dL between the supplementation group and non-supplemented groups and a standard deviation σ = 0.6 g/dL at post-intervention (adjusted to [31]), type 1 error probability at 0.05 (z_α/2_ = 1.96), statistical power at 90% (z_1−β_ = 1.28), and an assumed drop-out rate of 20%, a sample size of 420 was calculated.

Statistical analysis was performed using Microsoft Excel for Microsoft 365 MSO Version 2023 and the Statistical Package for Social Sciences, version 27.0 (SPSS Inc., Chicago, IL, USA). Diagrams were created with GraphPad Prism 8.0.1. Descriptive data were expressed as mean and standard deviation (mean ± SD), median and interquartile range (IQR) for metric, and percentage and number (% (*n*)) for categorical data. Normal distribution was evaluated with histograms and Q–Q-plots. Metric data were compared between groups by the Kruskal–Wallis test (posthoc test with adjustment by Bonferroni). Associations between metric data were analyzed by Kendall’s tau correlation. Categorical data were compared by Chi-square test and Fisher’s exact test at an expected cell count <5, and the effect size was displayed as Cramér’s V (data shown in supplementary material). Statistical significance was set at *p* < 0.05 (two-sided). Cases with incomplete datasets, for instance, due to refusal of single measures, were included with all available information and respective sample sizes per variable are provided.

## 3. Results

Of 471 recruited mother–infant pairs, 444 participated in the study at the third assessment. Mothers reporting termination of breastfeeding (*n* = 17) remained in the sample. As some women kept their appointments but with a delay, the time point of assessment ranged up to 8 months postpartum (one case at 2 months).

### 3.1. Nutritional Knowledge and Practice

The mothers achieved a mean *total* knowledge score of 0.41 (median 0.39). For the topics *three food group model*, *iron + anemia*, and *vitamin A + vitamin A deficiency*, on average, up to one-third of the questions were correctly answered, while for *malnutrition*, more than three-quarters were correctly answered (Table 2 and Tables S1–S4).

While two-fifths of the mothers knew at least one out of the three types of food, only 14.5% could recall all three of them, and around one-third knew foods belonging to one of these three types of food, respectively. Malnutrition was mostly defined as deficiency of nutrients or a disease. In all, 29.6% and 34.8% were familiar with the terms anemia or vitamin A deficiency. Mostly visible symptoms were stated, such as edema and skin/hair changes for malnutrition, pallor for anemia, and vision problems for vitamin A deficiency. Causes of malnutrition, anemia, and vitamin A deficiency were often related to low quantity or quality of food or nutrients as well as to disease. Preventive measures mentioned included eating diversified and nutrient-rich food, and the prevention or treatment of diseases. Green leafy vegetables were named as important sources for iron and vitamin A, and orange-fleshed vegetables as important sources for vitamin A. Less than 10% knew influencing factors on iron bioavailability. More mothers mentioned an incorrect answer regarding drinks decreasing iron bioavailability, mostly alcohol, than mentioned a correct answer.

The mean P-Score *iron* was 0.43 (median 0.50). Consumption of iron-rich foods was reported by around three-quarters, but the practice of fermentation and roasting of cereals/flour was hardly found.

### 3.2. Reasons for Dietary Choices and Attitudes towards Nutrition

Taste was stated most frequently as a reason for food selection and diversification (Table 3). Further frequent drivers were appearance, availability, and health (25.6–42.5%).

**Figure 2:**
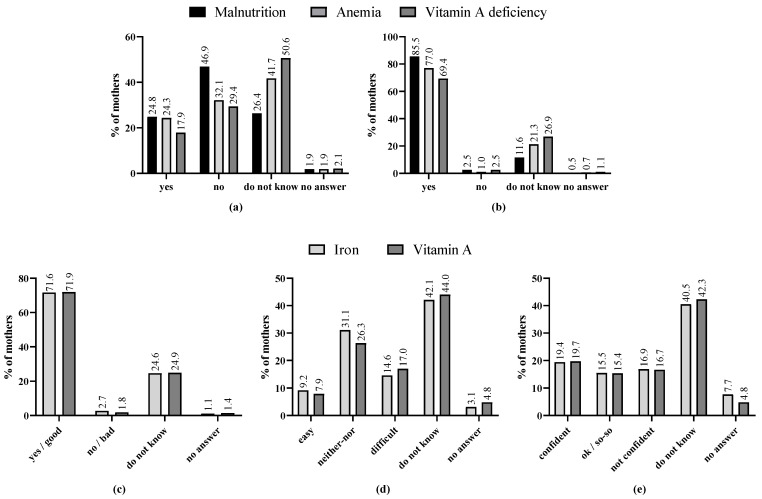
Nutrition-related attitudes of lactating mothers. Frequencies are displayed as percentages. (**a**) Perceived susceptibility to condition (*n* = 424, *n* = 420, *n* = 435), (**b**) Perceived severity of condition (*n* = 441, *n* = 418, *n* = 438), (**c**) Perceived benefits of nutrient-rich foods (*n* = 443, *n* = 442), (**d**) Perceived barriers to prepare nutrient-rich foods (*n* = 425, *n* = 441), (**e**) Self-confidence in preparing nutrient-rich foods (*n* = 439, *n* = 442).

More mothers considered it impossible that they themselves could be malnourished, anemic, or vitamin A deficient than possible; however, one-quarter to a half of the mothers could not give an estimation (Figure 2, Table S5). All three conditions were mostly seen as problematic. The preparation of iron- and vitamin A-rich foods was rated as beneficial, but around half of the mothers could not judge for themselves on barriers (difficulty) or confidence. Among those who considered being in a malnourished condition as impossible, significantly less mothers could state a reason for their self-perception compared with those rating it as possible (malnutrition: 62.1% vs. 86.7%, anemia: 34.4% vs. 49.5%, vitamin A deficiency: 29.3% vs. 68.4%; Table S6). Eating behavior or illness were stated as the main reasons for their estimation of susceptibility (Table S7). Among both mothers considering being malnourished as possible or not possible, some justified this estimation by the classification of malnutrition as a “disease” or as being “bad for health”. However, around one-fifth of the women considered susceptibility as a hypothetical situation in the case of certain nutritional or medical conditions, and did not consider it related to their actual situation (see Table S7). Severity of the malnutrition conditions were mostly based in their nature of a disease, harming the body, or being fatal. Furthermore, malnutrition was frequently called shameful. The positive value of iron- or vitamin A-rich foods was mostly justified by a general good effect on health. Some related them to construction or protection/immune system, blood (iron), or vision (vitamin A). Those rating preparation of iron- or vitamin A-rich foods as easy and being confident in it could justify this rating most frequently. Both difficulty and self-confidence in preparation were often related to financial means, availability, or knowledge, but also to the health effects of the foods.

### 3.3. Dietary Intake

**Figure 3:**
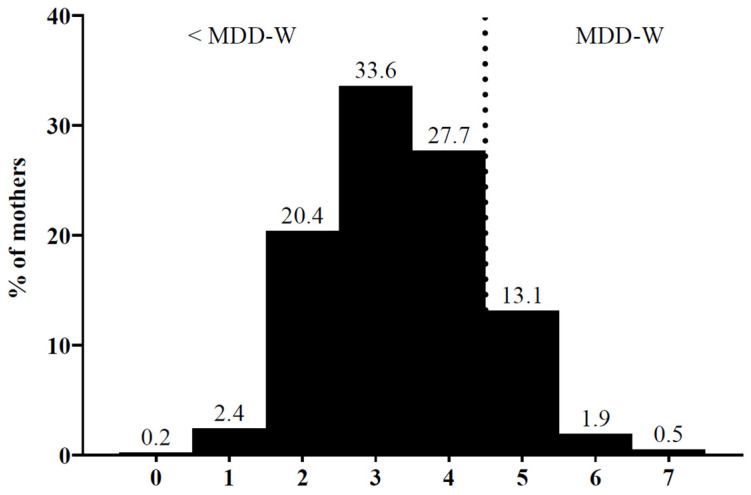
Distribution of Dietary Diversity Score achieved by lactating mothers. Frequencies are displayed as percentage (*n* = 411), MDD-W: Minimum Dietary Diversity for Women.

The Dietary Diversity Score (DDS) was low with a mean of 3.4 ± 1.1 (median 3.0, interquartile range 3.0–4.0) and ranging from zero to seven food groups. Only 15.6% reached MDD-W (Figure 3).

**Figure 4:**
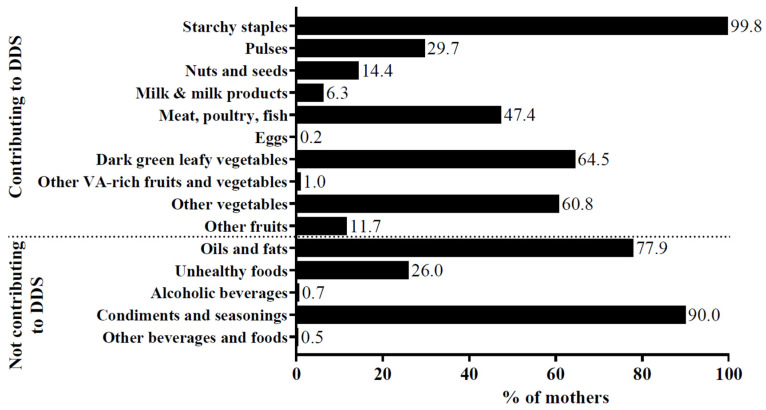
Consumption of food groups by lactating mothers. Frequencies are displayed as percentage (*n* = 411), DDS: Dietary Diversity Score, VA: vitamin A.

Starchy staples were consumed by nearly all women and dark green leafy vegetables and other vegetables by around 60% of women (Figure 4, Table S8). Fruit consumption was low. Meat, poultry, or fish were consumed by nearly half with fish being the main contributor, while the intake of other animal foods such as milk, dairy, and eggs was low. More than half of the mothers used palm oil. One-quarter consumed unhealthy foods, mainly self-prepared sugar-sweetened beverages or sugar added to other foods such as porridge.

### 3.4. Hemoglobin and Anemia

**Figure 5:**
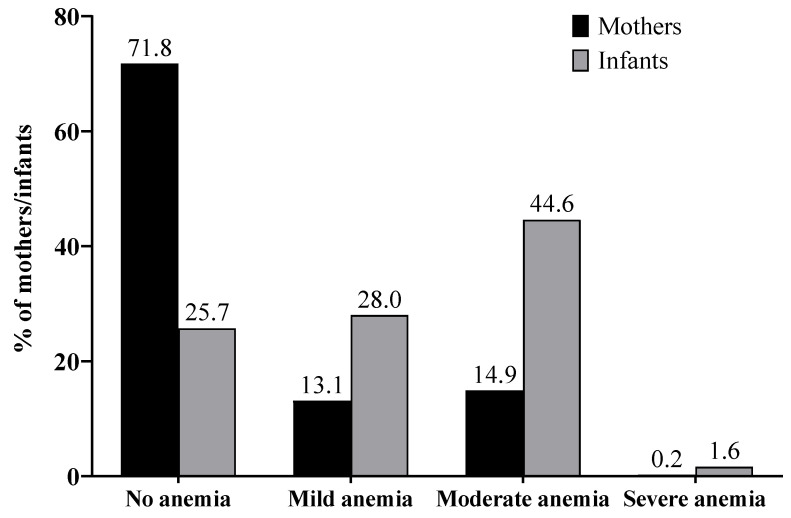
Anemia prevalence among mothers (*n* = 436) and infants (*n* = 439).

The mean Hb concentration of the mothers was 12.5 ± 1.5 g/dL (median 12.7 g/dL, interquartile range 11.7–13.5 g/dL), and 28.2 % suffered from anemia. Among the infants, the mean Hb was 10.1 ± 1.5 g/dL (median 10.1 g/dL, interquartile range 9.3–11.0 g/dL) with 74.3% being anemic (Figure 5).

### 3.5. Relationship of Nutritional Knowledge, Practice, Dietary Intake, and Hemoglobin Concentration

The K-Scores correlated significantly positively with each other, and the P-Score correlated negatively with the K-Score *vitamin A + vitamin A deficiency* (Table 4). A negative correlation was found between DDS and K-Score *vitamin A + vitamin A deficiency* and a slight positive, non-significant correlation was found between DDS and the K-Score *three food group model* (τ = 0.080, *p* = 0.052). Maternal Hb concentration was significantly positively related with the K-Scores *malnutrition*, *iron + anemia*, and *total*, and the P-Score *iron*. Maternal MUAC correlated positively with DDS, but neither MUAC nor DDS correlated significantly with Hb concentration. Maternal age did not correlate with K-/P-Scores, DDS, or Hb concentrations.

**Figure 6:**
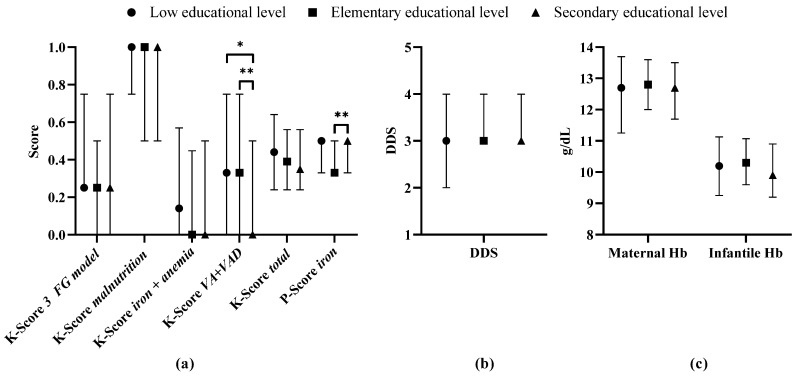
K-, P-Scores, DDS, and Hb concentration of lactating mothers by maternal educational level (low educational level: < 3 years at school: *n* = 71, elementary educational level: 3–6 years at school: *n* = 140, secondary educational level: ≥7 years at school: *n* = 230). Median, 25th and 75th percentile. (**a**) K- and P-Scores, (**b**) DDS, (**c**) Maternal and infantile Hb concentration. * Difference at *p* < 0.05; ** difference at *p* < 0.01 (Kruskal–Wallis test with posthoc adjusted by Bonferroni). *3 FG model*: *three food group model*, DDS: dietary diversity score, Hb: hemoglobin, K-Score: knowledge score, P-Score: practice score, *VA + VAD*: *vitamin A + vitamin A deficiency*.

K- and P-Scores, DDS, and Hb concentration were similar across educational levels, except K-Score *vitamin A + vitamin A deficiency* which was lower in mothers with secondary level education, and P-Score *iron*, which was lower among mothers with elementary level education. In the mean, K-Scores were highest at low educational level, except for *vitamin A + vitamin A deficiency*, and DDS increased slightly with increasing educational level, but these findings were not statistically significant (Figure 6, Table S9).

## 4. Discussion

### 4.1. Nutritional Knowledge

Nutritional knowledge was poor among mothers with most questions only being correctly answered by around one-third of them and a median *total* K-Score of 0.39 out of 1. Only in the section of *malnutrition* was greater knowledge present. This knowledge level is in line [15,16,32,33] or poorer [34,35,36] compared to studies in different African countries on different nutritional topics. Different study designs and knowledge topics covered may contribute to differing study results. According to the FAO’s threshold of an urgent need for nutrition education strategies at ≤70% correct answers to knowledge questions [2], nutrition education is crucial in the study population.

In this study, only around one-quarter to one-third of the mothers could mention symptoms, causes, or preventive measures of anemia or vitamin A deficiency or iron-/vitamin A-sources, respectively. Comparable [37] or better knowledge levels [34,38] on some of these topics were found in (pregnant) women of reproductive age or caregivers in other African countries. Furthermore, in our study, knowledge on more general information or visible symptoms was more pronounced than on the consequences of malnutrition on development or specific knowledge about nutrients and their deficiencies. This discrepancy suggests that malnutrition in general and inadequate energy intake is common in the region, so that mothers might be informed and know typical symptoms. Micronutrient deficiencies, that impair the development or immune system, may exist in the form of hidden hunger that caregivers are unaware of. Similar results were found by a qualitative study with pregnant women in DRC who presented good general knowledge on nutrition but poor knowledge about nutrients and their sources [39] and Tanzanian farmers [32].

### 4.2. Dietary Intake

The diet quality among the mothers in this study was poor with a median DDS of 3.0 (mean 3.4) and only 15.6% achieving MDD-W. In comparison with (pregnant/lactating) mothers and women of reproductive age in other African countries, this is at the bottom margin [16,33,36,40,41,42,43,44,45,46,47,48,49,50]. In lactating mothers or mothers of young children in Ethiopia and Zambia, 24.7–87.5% achieved MDD-W [36,40,41,42,43,44]. Differences between study results may be caused by the differing socio-demographic or economic status of mothers, knowledge, food security, or seasonality. The impact of seasonality is diminished in this study as the assessment covered both rainy and dry seasons.

Dietary practices towards iron intake were poor with a median P-Score of 0.50. Consumption of animal foods as well as dark green leafy vegetables was reported by a great part of the study participants in both the questionnaire and the 24hDR, suggesting frequent consumption. More than three-quarters reported their fruit consumption together with a meal. However, the low fruit consumption overall, reported in the 24hDRs, undermines the advantage of this beneficial time point. Over two-thirds stated that they consume coffee or tea with a meal; however, the 24hDRs revealed only low consumption. These discrepancies within the participants’ answers limit their explanatory power regarding beneficial or non-beneficial practices. Low usage of fermentation or roasting of flour was found in the study population, although fermentation of different foods is common among various African communities [51,52,53]. Indeed, 14.7% practiced fermentation, but the majority of them related it to manioc fermentation, which is part of the usual processing to remove cyanic acid [54]. The results suggest that, in addition to consumption of specific foods, knowledge and practice about food usage, such as processing methods, are poor and should be strengthened as a low-cost method for increasing nutrient bioavailability.

### 4.3. Anemia Status

Anemia prevalence was of moderate public health significance among the mothers (28.2%) and of severe public health significance among the infants (74.3%) [28]. Especially for the infants, this was considerably higher than reported in the last DHS 2013–2014 with 35.7% of the children aged 6–59 months being anemic in South Kivu and 22.7% of women of reproductive age [8]. However, while there are no age-specific anemia rates stated for South Kivu, for the whole DRC, the DHS reported higher anemia rates in the young age groups. This might contribute to the deviation between the overall number in South Kivu and in this study. There is a further discrepancy, with only 3.9% of the study participants self-reporting anemia diagnosis during pregnancy at the baseline of this study [14]. This suggests a potentially high amount of undetected micronutrient deficiencies in the population. The lack of an association of Hb with MUAC indicates occurrence of anemia independent from underweight.

Other studies in South Kivu found lower rates of anemia in non-pregnant women of reproductive age (16.5%) and children aged 6–23 months (47.8–58.6%) compared to our findings [9,10,11]. Rates of iron deficiency were lower than rates of anemia, but frequent infections and genetic Hb disorders were mentioned as potential influencers. Determining iron deficiency by free erythrocyte protoporphyrin resulted in higher levels of iron deficiency [9]. Factors associated with anemia were malnutrition, illness, *Plasmodium* infection, and zinc and partly iron deficiency, when not measured by serum ferritin [9,10]. Bahizire et al. [10] suggested high iron concentrations in the drinking water due to the volcanic soil in the Kivu region as a possible reason for rare iron deficiency.

An analysis of DHS data from 46 low- and middle-income countries reported an anemia prevalence of 50.95% among lactating mothers, with a wide range from 11.71% to 60.49% [6]. In nine East-African countries, prevalence ranged from 19.33% to 53.08% with a mean of 36.15% [18]. A pooled analysis of 32 Sub-Saharan African countries’ DHS data found an anemia prevalence of 76.1% among children aged 6–23 months [55] which is close to the prevalence of anemia found among infants aged 3–8 months in our study (74.3%).

Considering the reported results of other studies in South Kivu and the quite high dietary intake of iron-rich animal-sourced foods and dark green leafy vegetables, the anemia rates observed in this study may only be partly attributed to iron deficiency. Other reasons may include hemoglobinopathies, malaria, or helminth infections [9,10,21,29,56,57,58,59,60,61], but also malnutrition in terms of other nutrients such as zinc, vitamin A, B12, or folate [9,21,29,57]. The latter will be discussed in the next section. Malaria was the most commonly reported health problem during pregnancy and there was no full coverage of antimalarial and antihelminthic treatment during pregnancy in this study cohort [14].

### 4.4. Relationship of Nutritional and Socio-Demographic Factors

Nutritional knowledge was not significantly positively correlated with dietary practices. Surprisingly, the K-Score *vitamin A + vitamin A deficiency* was negatively associated with the P-Score *iron* and the DDS. The reasons for this finding are unclear. There was a slight positive, but non-significant association of DDS with the K-Score *three food group model*. The K- and P-Scores *iron* were not related. Also, studies in Africa [33,35,40,43,47,62,63,64] and Asia [33,65,66,67] and a review of studies in mainly industrialized countries [68] mostly found positive, often weak associations of nutritional knowledge/having received nutrition information with dietary intake, dietary diversity, food variety, or consumption of animal foods among (pregnant) mothers, caregivers, and the community.

The P-Score *iron* was higher than the respective K-Score. Dark green leafy vegetables, palm oil, and flesh foods were frequently consumed, while the knowledge about their nutrient content was low. Thus, their consumption is suggested to be based on other reasons such as habitude, traditional uses and beliefs, and availability, as well as affordability. The frequent use of palm oil is in line with the results of Moumin et al. [12] who found red palm oil as a main contributor of vitamin A intake in women of reproductive age in South Kivu. Dark green leafy vegetables are a common food in several African countries [42,47,49,69]. The consumption of meat, poultry, and fish was unexpectedly high (47.4%) in comparison to studies on (lactating) mothers and women of reproductive age in Malawi [49], Tanzania [46,63], Kenya [45], and Ethiopia [33,40,41,47] (4.6–42.2%). It could be attributed to the Bukavu’s location at the lake Kivu and, thus, the availability of fresh and dried fish, which was the main contributor within the food group. However, studies on mothers in Zambia [42], Ethiopia [36], and Ghana [69] reported even higher intakes (up to 72.1% for fish). These foods provide several nutrients with an impact on Hb concentration such as iron, zinc, vitamin A, B12, or folate [57,70]. On the other hand, a high intake of starchy staples was reported in the 24hDRs. Cereals have a high phytate content that reduces the bioavailability of iron and zinc in the diet [70]. Practices enhancing bioavailability, such as consuming fruits with a meal, were hardly reported. The low dietary diversity overall suggests an inadequate micronutrient intake [27,71,72]. Altogether, these practices contribute to higher anemia risk.

We found no association of maternal Hb with DDS, but slight, positive correlations with some K-Scores including *iron + anemia* and the P-Score *iron.* Similar results were reported for pregnant women in Ghana [73], while in postpartum mothers both higher knowledge and MDD-W were associated with lower anemia risk [74]. Evidence on an association of DDS/MDD-W with Hb or anemia risk in (pregnant/postpartum) women of reproductive age is conflicting [50,73,74,75,76,77,78]. Potential relationships and causality deserve further examination. Nutritional knowledge might have beneficial impacts on nutritional status by influencing dietary practices and portion sizes consumed that are not reflected in the DDS.

On the other hand, the DDS was significantly associated with maternal MUAC. Although dietary diversity is used as an indicator of micronutrient adequacy [27], consumption of a higher number of food groups may be associated with higher total food and, thereby, energy intake. It can be further interpreted as a sign of a potential double burden of underweight and micronutrient deficiency in the study population.

Self-reported drivers of food selection and diversification were multifaceted, including taste, appearance, health effects, and availability of foods. The price was mentioned by only around one-tenth of the mothers. However, it has been observed that formulations referring to availability were often used with the meaning of affordability; thus, overlaps are possible. Similarly, women in South Africa [79], Rwanda [80], Ghana [81], and Ethiopia [82] reported taste, health issues, food price, affordability, freshness of food, and family diet, or poverty, food production, food preferences, and food characteristics such as taste as drivers or constraints for dietary behavior, among others.

Availability and affordability of foods may hinder the translation of nutritional knowledge into practice and subsequent nutritional status. They were frequently mentioned as reason for the self-perceived barriers or confidence in preparing nutrient-rich foods and might contribute to the lack of associations between knowledge and practices. Others found economic factors more important for dietary quality than nutritional knowledge [16,62]. The high prevalence of food insecurity in DRC population (69.2% in 2018–2020) [83] might contribute to the low DDS observed in this study. In Ethiopian pregnant women, food security, production of own crops, and better nutritional knowledge and attitudes have been found to be associated with dietary adequacy [64], resembling the motives of availability, affordability, and promoting health in this study.

While most mothers rated the value of iron or vitamin A as good and the respective deficiency as bad, nearly each second mother could not give a self-estimation about her health status and barriers and confidence in preparation of iron- or vitamin A-rich foods. These findings are in line with the low knowledge levels, and it can be interpreted that the mothers lacked the knowledge base to evaluate their own situation. Furthermore, maternal nutrition-related attitudes were often unfounded, especially among those considering themselves not as potentially malnourished, and several mothers called malnutrition “a disease”. Both factors may indicate limited understanding of the relationship of dietary intake with health status, potentially leading to an unfavorable dietary intake. As health effects of foods were frequent drivers of food selection, increasing knowledge is crucial. Furthermore, non-visible symptoms of malnutrition, especially anemia and vitamin A deficiency, were hardly known. This could contribute to unfounded self-perceptions of their own nutritional status. Malnutrition was seen as shameful by several mothers, which might limit realistic self-perception as well as health-seeking behavior for treatment. The importance of nutrition-related attitudes is confirmed by studies that found associations of nutritional knowledge with attitudes [15,34], of combined knowledge and attitudes score with household dietary diversity [35], and of knowledge, attitudes, and practices with each other [84].

There was no relationship between K-/P-Scores, DDS, or Hb and maternal age. Some K-Scores, especially *vitamin A + vitamin A deficiency*, tended to be lower with high educational level, while the P-Score was lowest in women with elementary level. The slight inverse relation of nutritional knowledge and formal education suggests that nutritional information is not regularly provided in schools but rather in other institutions such as health centers. Other authors who found lower nutritional knowledge, but better dietary practices, with rising maternal education related their findings to the focus of nutrition education on low-income levels [16]. On the other hand, mothers of higher age tend to have delivered more children and should have received health and nutrition information more often in ante- and postnatal care. Lack of associations may be attributed to the fact that nutrition is not the main focus in these sessions or that mothers do not retain the information in the long-term.

DDS rose slightly with higher educational level, though it was not statistically significant. Better education may be associated with higher socio-economic status allowing for a more diverse diet. Other studies found higher dietary quality associated with higher educational level [15,17,40,43,45,85,86,87] and age [17,86,87,88]. Also, in contrast to our study, hemoglobin concentration or anemia risk were found to be correlated with educational level [6,7,18,76,89,90] and age [6,7,18,90]. Some other studies did not find this relationship, either [73,91].

### 4.5. Limitations

The findings need to be interpreted in the light of some limitations. Due to the inclusion criteria of the study, the study population is not representative. There was just one 24hDR per person per assessment, which is why groups instead of individuals need to be evaluated [27] and correlation analyses should be interpreted with caution. Portion sizes needed partly to be estimated by standard portions or by taking into account all consumers of a meal for side ingredients. If meals were not prepared by the respondents themselves, side ingredients could not always be stated, leading to a potential underestimation. However, preparation by others was rarely reported and side ingredients had often been added in small amounts, limiting the potential bias. Furthermore, due to the difficulties in portion size estimation, the dietary diversity score was used as it does not rely on portion size except for the border of 15 g. Despite training, interviewer bias could have occurred, especially for 24hDRs. Genetic factors and infectious diseases that might contribute to anemia, dietary intake of infants, as well as data on household food security or wealth were not assessed in this study and should be evaluated in the region. Based on the cross-sectional analysis, no conclusions on causality can be drawn.

## 5. Conclusions

This study highlights poor nutritional knowledge and practices among lactating mothers around Bukavu, and moderate to high anemia rates among mothers and infants. Self-reported drivers of dietary choices were multifaceted, including taste, availability, affordability, and health effects of foods. These various personal and environmental determinants need to be addressed in a holistic approach to promote beneficial food choices and subsequently, nutritional status. Formal education did not seem to play a main role, yet, thus the inclusion of schools in nutritional interventions should be promoted. There were only slight, partly non-significant associations between knowledge, dietary intake, and hemoglobin concentration. A great number of the mothers stated unfounded self-perceptions about their own health status and nutrient-rich foods. This may result in unfavorable dietary choices and poor seeking for health care in case of disease symptoms. Thus, increasing nutritional knowledge is crucial in overcoming this risky behavior. Knowledge and awareness about micronutrient deficiencies and anemia were especially rare and need to be improved. Research is needed to explore potential causality between knowledge, attitudes, practices, and nutritional status as well as to better understand further contributing factors.

This cross-sectional analysis was followed by an intervention study and qualitative assessments for a deeper insight and evaluation of nutrition-specific interventions. The presented assessment on nutritional knowledge and practices could have raised awareness on nutrition among the study participants. However, any potential effect would have taken place in all intervention and control groups, thereby not biasing the intervention study. The results will be published elsewhere.

## Figures and Tables

**Table 1:** Questions included in K- and P-Scores.

K-Score *Three Food Group* Model	K-Score *Malnutrition*	K-Score *Iron* + *Anemia*	K-Score *Vitamin A* + *Vitamin A Deficiency* (VAD)	P-Score Iron
Can state at least 1 of the 3 types of food *	Can state a definition of malnutrition	Can state a sign/symptom of anemia	Can state a sign/symptom of VAD	Consumption of animal foods ≥ 1×/week
Can state an energy-giving food	Can state a consequence of malnutrition	Can state a consequence of iron deficiency in children		Consumption of green leafy vegetables ≥ 3×/week
Can state a constructive food	Can state a cause of malnutrition	Can state a cause of anemia	Can state a cause of VAD	Consumption of fruits with a meal
Can state a protective food	Can state a preventive measure for malnutrition	Can state a preventive measure for anemia	Can state a preventive measure for VAD	Consumption of coffee/tea only outside a meal
		Can state an iron-rich food	Can state a vitamin A-rich food	Fermentation of flour
		Can state a method increasing iron bioavailability		Roasting of grains
		Can state a drink reducing iron bioavailability		

* 3 types of food: energy-giving, constructive, protective. Terms in italics describe the titles of the respective K- and P-Score. K-Score: knowledge score, P-Score: practice score, VAD: vitamin A deficiency.

**Table 2:** Knowledge and practices of lactating mothers, prevalence and scores (*N* = 444).

Variables ^a,b^	% (*n*)	Score
K-Score *three food group model* (*n* = 438)		0.36 ± 0.35 0.25 (0.00, 0.75)
Knows at least one of the 3 types of food (*n* = 435)	41.1 (179)	
Knows an energy-giving food (*n* = 437)	39.1 (171)	
Knows a constructive food (*n* = 439)	33.7 (148)	
Knows a protective food (*n* = 436)	29.4 (128)	
K-Score *malnutrition* (*n* = 441)		0.77 ± 0.33 1.00 (0.50, 1.00)
Can define malnutrition (*n* = 436)	70.6 (308)	
Knows a consequence/symptom of malnutrition (*n* = 441)	85.9 (379)	
Knows a cause of malnutrition (*n* = 441)	79.1 (349)	
Knows a measure to prevent malnutrition (*n* = 439)	70.8 (311)	
K-Score *iron + anemia* (*n* = 428)		0.24 ± 0.31 0.00 (0.00, 0.50)
Knows a sign/symptom of anemia (*n* = 438) ^c^	27.2 (119)	
Knows a consequence of iron-deficient nutrition in children (*n* = 438)	23.3 (102)	
Knows a cause of anemia (*n* = 440)	33.0 (145)	
Knows a measure to prevent anemia (*n* = 440)	32.5 (143)	
Knows an iron-rich food (*n* = 439)	32.3 (142)	
Knows a food/method increasing iron bioavailability (*n* = 438)	7.8 (34)	
Knows a drink decreasing iron bioavailability (*n* = 434)	4.1 (18)	
K-Score *vitamin A + vitamin A deficiency* (*n* = 435)		0.33 ± 0.40 0.00 (0.00, 0.75)
Knows a sign/symptom of vitamin A deficiency (*n* = 438) ^c^	22.4 (98)	
Knows a cause of vitamin A deficiency (*n* = 439)	35.8 (157)	
Knows a measure to prevent of vitamin A deficiency (*n* = 439)	30.1 (132)	
Knows a vitamin A-rich food (*n* = 437)	34.3 (150)	
K-Score *total* (*n* = 439)		0.41 ± 0.24 0.39 (0.24, 0.56)
P-Score *iron* (*n* = 425)		0.43 ± 0.14 0.50 (0.33, 0.50)
Eats fruits with a meal (*n* = 441)	78.5 (346)	
Does not drink coffee/tea with a meal (*n* = 440)	31.1 (137)	
Practices fermentation of flour (*n* = 421)	1.0 (4)	
Practices roasting of flour (*n* = 441)	2.9 (13)	
Eats animal foods at least once per week (*n* = 425)	76.7 (326)	
Eats green leafy vegetables at least 3× per week (*n* = 442)	73.3 (324)	

^a^ Categorical variables are expressed as % (*n*) and metric variables are expressed as mean ± SD and median (IQR). ^b^ Lack of corresponding sum of frequencies with total sample size is due to missing data; total frequencies per variable are given. ^c^ Not knowing anemia/vitamin A deficiency was considered as not knowing a symptom. Terms in italics describe the titles of the respective K- and P-Score.

**Table 3:** Self-reported reasons for food selection of lactating mothers (*N* = 444).

Variables ^a,b^	% (*n*)
Reasons for food choice (*n* = 442) ^c^	
Taste	68.1 (301)
Appearance	42.5 (188)
Good for Health	29.0 (128)
Availability	25.6 (113)
Price	12.2 (54)
Do not select	0.9 (4)
Availability/affordability	0.5 (2)
Diversified meal	0.5 (2)
According to plans	0.2 (1)
Do not know	1.8 (8)
No answer	0.7 (3)
Reasons for food diversification (*n* = 441) ^c^	
Taste	49.9 (220)
Good for health	40.4 (178)
Appearance	39.5 (174)
Availability	33.8 (149)
Price	10.0 (44)
For balancing, diversifying	0.7 (3)
For satisfaction	0.5 (2)
Habit	0.2 (1)
Insufficient meal without	0.2 (1)
Do not know	2.7 (12)
No answer	0.7 (3)

^a^ Categorical variables are expressed as % (*n*). ^b^ Lack of corresponding sum of frequencies with total sample size is due to missing data; total frequencies per variable are given. ^c^ Multiple response question.

**Table 4:** Correlation of knowledge and practice scores of lactating mothers.

Variables		K-Score *3 FG Model*	K-Score *Malnutrition*	K-Score *Iron + Anemia*	K-Score *Vitamin A + VAD*	K-Score *Total*	P-Score *Iron*	Dietary Diversity Score	Maternal Hb	Infant Hb
K-Score *malnutrition*	τ	**0.292**								
	*p*-value ^b^	**<0.001**								
	*n* ^a^	437								
K-Score *iron + anemia*	τ	**0.313**	**0.231**							
	*p*-value ^b^	**<0.001**	**<0.001**							
	*n* ^a^	424	427							
K-Score vitamin A *+ VAD*	τ	**0.256**	**0.185**	**0.301**						
	*p*-value ^b^	**<0.001**	**<0.001**	**<0.001**						
	*n* ^a^	430	433	420						
K-Score *total*	τ	**0.562**	**0.523**	**0.615**	**0.496**					
	*p*-value ^b^	**<0.001**	**<0.001**	**<0.001**	**<0.001**					
	*n* ^a^	437	439	425	431					
P-Score *iron*	τ	0.023	−0.004	−0.028	**−0.093**	−0.030				
	*p*-value ^b^	0.575	0.931	0.492	**0.027**	0.425				
	*n* ^a^	421	424	413	417	422				
Dietary Diversity Score	τ	0.080	0.055	0.025	**−0.091**	0.039	−0.011			
	*p*-value ^b^	0.052	0.186	0.552	**0.029**	0.303	0.791			
	*n* ^a^	405	408	396	403	406	393			
Maternal Hb	τ	0.053	**0.090**	**0.086**	0.066	**0.095**	**0.085**	−0.009		
	*p*-value ^b^	0.140	**0.015**	**0.018**	0.073	**0.004**	**0.023**	0.814		
	*n* ^a^	430	433	420	427	431	419	404		
Infant Hb	τ	0.001	0.003	0.001	0.007	0.003	0.001	0.012	0.027	
	*p*-value ^b^	0.971	0.938	0.987	0.846	0.939	0.986	0.749	0.419	
	*n* ^a^	433	436	423	430	434	421	409	433	
Maternal MUAC	τ	0.034	0.001	−0.007	−0.028	0.004	0.001	**0.121**	0.057	0.059
	*p*-value ^b^	0.334	0.987	0.848	0.439	0.897	0.972	**0.001**	0.081	0.069
	*n* ^a^	437	440	427	434	438	424	410	436	439
Maternal age	τ	−0.009	−0.006	−0.020	0.000	−0.005	−0.055	0.047	−0.059	0.021
	*p*-value ^b^	0.803	0.864	0.592	0.996	0.880	0.148	0.218	0.079	0.531
	*n* ^a^	427	430	419	424	428	415	400	425	428

^a^ Lack of corresponding sum of frequencies with total sample size is due to missing data; total frequencies per variable-pair are given. ^b^ Significantly different at *p*-value < 0.05 (in bold); *p*-value was derived using Kendall’s tau correlation. Terms in italics describe the titles of the respective K- and P-Score. *3 FG model*: *three food group model*, Hb: hemoglobin, K-Score: knowledge score, MUAC: mid-upper arm circumference, P-Score: practice score, VAD: vitamin A deficiency.

## Data Availability

The data presented in this study are available on request from the corresponding author due to ongoing analysis and paper writing.

## Supplementary Materials

The following supporting information can be downloaded at: https://www.mdpi.com/article/10.3390/nu16060870/s1, Table S1: Knowledge of lactating mothers about the three food group model and malnutrition; Table S2: Knowledge of lactating mothers about iron and anemia; Table S3: Knowledge of lactating mothers about vitamin A and vitamin A deficiency; Table S4: Practices of lactating mothers regarding iron; Table S5: Nutrition-related attitudes of lactating mothers; Table S6: Ability of justification for nutrition-related attitudes of lactating mothers; Table S7: Nutrition-related attitudes and associated reasons of lactating mothers; Table S8: Food group consumption of lactating mothers; Table S9: K-, P-Scores, DDS, and Hb concentration of lactating mothers and infants by maternal educational level.
