# Supplementary material for: Nutrition-Related Knowledge, Attitudes, Practices, and Anemia Status of Lactating Mothers in Bukavu, Democratic Republic of the Congo—A Cross-Sectional Analysis
Source: Nutrients. 2024 Mar 17;16(6):870. doi: 10.3390/nu16060870 (PMC10975608; doi:10.3390/nu16060870)
Supplement: Supplementary file 1 [file nutrients-16-00870-s001.zip › Supplementary tables_Knowledge, attitudes, practices, anemia status among lactating mothers in DRC.pdf]

Table S1: Knowledge of lactating mothers about the three food group model and malnutrition

**Variables** [a]	**Total** [b] *N*=444
Knowledge about three food group model	
Good Food[c]	*n*=434
Three food group model	30.6 (133)
Other	40.3 (175)
Do not know	29.3 (127)
Three food group model[c]	*n*=435
Energy-giving	31.5 (137)
Construction	27.1 (118)
Protection	25.3 (110)
Wrong answer	9.4 (41)
Do not know	50.1 (218)
Three food group model – Number of correct answers	*n*=435
0	58.9 (256)
1	12.9 (56)
2	13.8 (60)
3	14.5 (63)
Energy-giving foods[c]	*n*=437
Staples	15.8 (69)
Oil	7.1 (31)
Sugar / sugared foods/drinks	24.3 (106)
Other correct answer	1.6 (7)
Wrong answer	19.9 (87)
Do not know	49.0 (214)
Constructive foods[c]	*n*=439
Animal foods	17.1 (75)
Legumes	23.7 (104)
Wrong answer	15.3 (67)
Do not know	56.9 (250)
Protective foods[c]	*n*=436
Vegetables	22.0 (96)
Fruits	15.4 (67)
Wrong answer	10.8 (47)
Do not know	63.8 (278)
Knowledge about malnutrition	
Malnutrition[c]	*n*=436
Deficiency of nutrients	44.3 (193)
Bad life	0.7 (3)
Change of the body	1.1 (5)
Poor diet, lack of food/nutrients	4.1 (18)
Disease	19.3 (84)
Disease due to poor diet	0.2 (1)
Disease due to short birth space	0.2 (1)
Edema, swelling of cheeks and feet	0.5 (2)
Impaired health, decrease of vitamins	0.2 (1)
Intestinal worms	0.9 (4)
Lack of means	0.2 (1)
Weakness	0.5 (2)
Other, not precised	0.7 (3)
Do not know	28.7 (125)

Consequences and symptoms of malnutrition[c]	*n*=441
Weakness, lack of energy to work	13.4 (59)
Get infectious diseases (more easily)	1.8 (8)
Loss of weight, face of an old person	10.2 (45)
Impaired mental and physical development	4.3 (19)
Edema	50.8 (224)
Change of the skin	65.5 (289)
Change of hair	70.3 (310)
Change of the body	1.8 (8)
Death	0.2 (1)
Consequences of iron deficiency	0.1 (1)
Intestinal worms	0.9 (4)
Lack of appetite	0.2 (1)
Do not know	14.1 (62)
Causes of malnutrition[c]	*n*=441
Eating too little	46.0 (203)
Eating too watery meals (poor in nutrients)	16.3 (72)
Eating non-divers meals (monotony)	14.3 (63)
Not eating during illness	10.2 (45)
Parasitic infections	1.4 (6)
Poor breastfeeding practices	0.2 (1)
Disease	1.4 (6)
Poor diet	11.6 (51)
Bad life	0.7 (3)
Pregnancy while small infant/breastfed infant	0.5 (2)
Heredity	0.2 (1)
Lack of consideration in the family	0.2 (1)
Lack of financial means, poverty	1.1 (5)
Lack of peace	0.5 (2)
Weaning	0.2 (1)
Wrong answer	0.2 (1)
Do not know	20.9 (92)
Prevention of malnutrition[c]	*n*=439
Eating diversified food	28.0 (123)
Eating nutrient-rich food	34.2 (150)
Avoiding short birth space	0.2 (1)
Child care	0.2 (1)
Consulting medical staff, going for treatment	2.7 (12)
Eating enough / a lot	4.6 (20)
Deworming	0.2 (1)
Good food, eating, eating well / regularly, preparing well	12.5 (55)
Hygiene (food, body) / cleanliness	0.9 (4)
Knowledge	0.5 (2)
Living well	0.2 (1)
Financial means, work	1.8 (8)
Wrong answer	0.2 (1)
Do not know	29.2 (128)

[a] Categorical variables are expressed as % (n).

[b] Lack of corresponding sum of frequencies with total sample size is due to missing data; total frequencies per variable are given.

[c] Multiple response question.

Table S2: Knowledge of lactating mothers about iron and anemia

Variables [a]	Total [b] *N*=444
Iron, iron deficiency, anemia	
Knowing iron	*n*=434
Yes	4.8 (21)
No	86.4 (375)
Do not know	38 (8.8)
Knowing anemia	*n*=442
Yes	29.6 (131)
No	61.5 (272)
Do not know	8.8 (39)
Signs/symptoms of anemia[c]	*n*=438
Fatigue, weakness, lack of energy	11.9 (52)
Pallor	20.3 (89)
Spoon-shaped nails	2.3 (10)
Getting infectious diseases (more easily)	2.5 (11)
Change of the skin	0.2 (1)
Vertigo	1.6 (7)
Feeling cold	0.2 (1)
Wrong answer	0.2 (1)
Do not know	1.8 (8)
Does not know anemia	71.0 (311)
Consequences of iron-deficient nutrition in children[c]	*n*=438
Impaired growth	10.3 (45)
Impaired mental and physical development	9.1 (40)
(Frequent) illness	2.1 (9)
Decrease of blood, anemia	0.7 (3)
Death	3.2 (14)
Poor health	0.2 (1)
Vertigo	0.2 (1)
Weakness	1.1 (5)
Intestinal worms	0.2 (1)
Lack of appetite	0.2 (1)
Wrong answer	1.6 (7)
Do not know	75.1 (329)
Causes of anemia[c]	*n*=440
Food with low iron content	6.4 (28)
Eating too little	12.5 (55)
Diseases, infections	17.3 (76)
Poor diet (quantiy/quality, not precised)	3.6 (16)
Blood loss	0.2 (1)
Do not know	67.0 (295)
Prevention of anemia[c]	*n*=440
Eating iron-rich foods	14.5 (64)
Eating vitamin C-rich foods with/after meals	5.7 (25)
Ferment, roast, grind, soak to germinate	1.4 (6)
Prevent/treat diseases/infections – visit hospital/health center	16.6 (73)
Eating blood-building foods	0.2 (1)
Eating enough/well	0.9 (4)
Eating foods enhancing iron bioavailability, nutrient-rich	0.5 (2)
Hygiene	0.2 (1)
Testing	0.2 (1)
Wrong answer	0.9 (4)
Do not know	67.5 (297)

Iron-rich foods[c]	*n*=439
Organs	3.9 (17)
Meat, poultry	1.6 (7)
Fish	1.8 (8)
Insects	0.9 (4)
Green leaves	28.7 (126)
Legumes	3.2 (14)
Seeds, nuts	0.7 (3)
Other correct foods	0.7 (3)
Wrong answer	2.3 (10)
Do not know	67.4 (296)
Foods and methods increasing iron bioavailability[c]	*n*=438
Vitamin C-rich foods, especially fruits	4.6 (20)
Potatoes, tomatoes, cabbage	1.1 (5)
Small quantity of meat/fish	2.7 (12)
Fermentation	0.5 (2)
Roasting	1.1 (5)
Grinding	0.5 (2)
Soaking to germinate	0.2 (1)
Other correct food/method	0.5 (2)
Wrong answer	0.7 (3)
Do not know	91.8 (402)
Drinks decreasing iron bioavailability[c]	*n*=434
Coffee	3.5 (15)
Tea	0.2 (1)
Milk	0.9 (4)
Wrong answer	8.1 (35)
Do not know	88.7 (385)
Knowing fermentation	*n*=435
Yes	36.6 (159)
No	60.9 (265)
Do not know	2.5 (11)

[a] Categorical variables are expressed as % (n).

[b] Lack of corresponding sum of frequencies with total sample size is due to missing data; total frequencies per variable are given.

[c] Multiple response question.

Table S3: Knowledge of lactating mothers about vitamin A and vitamin A deficiency

**Variables** [a]	**Total** [b] ***N*=444**
Vitamin A, vitamin A deficiency (VAD)	
Knowing vitamin A deficiency (VAD)	*n*=440
Yes	34.8 (153)
No	58.9 (259)
Do not know	6.4 (28)
Signs/symptoms of VAD[c]	*n*=438
Weakness, feeling less energetic	12.8 (56)
Getting infectious diseases (more easily)	4.1 (18)
Problems of vision: nightblindess, dry eyes, damage of the cornea, blindness	9.8 (43)
Wrong answer	1.4 (6)
Do not know	10.7 (47)
Does not know VAD	65.5 (287)
Causes of VAD[c]	*n*=439
Food with low vitamin A content	14.4 (63)
Eating without diversity of meals	8.0 (35)
Eating too little	10.0 (44)
Poor diet	3.4 (15)
Disease	2.5 (11)
Lack of vitamin A	0.2 (1)
Lack of means	0.2 (1)
Not going for treatment	0.2 (1)
Wrong answer	0.7 (3)
Do not know	63.6 (279)
Prevention of VAD[c]	*n*=439
Eating vitamin A-rich foods	21.6 (95)
Good diet (eating, eating enough/a lot/normally/regularly/well/everything)	6.6 (29)
Consulting medical staff with problem, treat diseases	0.9 (4)
Enough financial means	0.5 (2)
Eating diversified food	0.5 (2)
Eating recommended food / vitamin A-rich food if known	0.5 (2)
Food hygiene	0.5 (2)
Eating blood-building food	0.2 (1)
Fruits	0.2 (1)
Vitamin A supplementation	0.2 (1)
Increase vitamin A	0.2 (1)
Wrong answer	0.5 (2)
Do not know	69.7 (306)
Vitamin A-rich foods[c]	*n*=437
Organs	3.9 (17)
Egg/egg yolk	2.7 (12)
Fish	3.2 (14)
Milk, yogurt, cheese	4.1 (18)
Green leaves	14.6 (64)
Orange-fleshed vegetables	17.8 (78)
Orange-fleshed fruits	6.2 (27)
Palm oil	2.5 (11)
Wrong answer	1.6 (7)
Do not know	64.8 (283)

[a] Categorical variables are expressed as % (n).

[b] Lack of corresponding sum of frequencies with total sample size is due to missing data; total frequencies per variable are given.

[c] Multiple response question.

VAD: vitamin A deficiency.

Table S4: Practices of lactating mothers regarding iron

**Variables** [a]	**Total** [b] *N***=444**
Iron-rich foods, bioavailability	
Time point of eating fruits[c]	*n*=441
Directly before the meal	11.8 (52)
With the meal	10.7 (47)
Directly after the meal	66.6 (293)
Between the meals	9.3 (41)
No specific time given	12.7 (56)
Do not know	2.5 (11)
Drinking coffee or tea	*n*=441
Yes	75.7 (334)
No	24.0 (106)
Do not know	0.2 (1)
Time point of drinking coffee/tea, if drinking[c]	*n*=333
Directly before the meal	18.6 (62)
With the meal	14.4 (48)
Directly after the meal	74.5 (248)
Between the meals	9.3 (31)
No specific time given	4.5 (15)
Practicing fermentation	*n*=423
Yes	14.7 (62)
No	82.0 (347)
Do not know	3.3 (14)
Fermented foods, if practicing fermentation[d]	*n*=62
Manioc	77.4 (48)
Avocado	11.3 (7)
Banana	6.5 (4)
Manioc for *Cikwange* (local food with fermented manioc)	6.5 (4)
Wheat	4.8 (3)
Maize	1.6 (1)
All	1.6 (1)
Fruits	1.6 (1)
Vegetables	1.6 (1)
Mandale	3.2 (2)
Practicing roasting of flour	*n*=441
Yes	2.9 (13)
No	95.5 (421)
Do not know	1.6 (7)
Consumption frequency of animal foods	*n*=425
Several times per day	0.2 (1)
Once per day	2.8 (12)
5-6x per week	5.2 (22)
3-4x per week	16.5 (70)
2x per week	30.6 (130)
Once per week	21.4 (91)
Once per 2 weeks / 2x per month	8.0 (34)
Once per month	5.4 (23)
Rarely	0.9 (4)
Never	5.2 (22)
According to financial availability	2.1 (9)
At any time	0.2 (1)
Do not eat meat and milk	0.2 (1)
Do not know	1.2 (5)

Consumption frequency of green leafy vegetables	*n*=442
Several times per day	10.9 (48)
Once per day	24.2 (107)
5-6x per week	18.1 (80)
3-4x per week	20.1 (89)
2x per week	19.5 (86)
Once per week	3.8 (17)
Once per 2 weeks / 2x per month	1.6 (7)
Once per month	0.7 (3)
Eating by lack of means	0.2 (1)
According to availability	0.2 (1)
Causing amoeba	0.2 (1)
Satisfaction	0.2 (1)
Do not know	0.2 (1)

[a] Categorical variables are expressed as % (n).

[b] Lack of corresponding sum of frequencies with total sample size is due to missing data; total frequencies per variable are given.

[c] Multiple response question.

Table S5: Nutrition-related attitudes of lactating mothers

Variables [a]	Total [b] *N*=444
Attitudes towards malnutrition	
Perceived susceptibility: Possibility of being malnourished	*n*=424
Possible	24.8 (105)
Not possible	46.9 (199)
Do not know	26.4 (112)
No answer	1.9 (8)
Perceived severity: Evaluation of malnutrition	*n*=441
Problem	85.5 (377)
No problem	2.5 (11)
Do not know	11.6 (51)
No answer	0.5 (2)
Attitudes towards anemia	
Perceived susceptibility: Possibility of being anemic	*n*=420
Possible	24.3 (102)
Not possible	32.1 (135)
Do not know	41.7 (175)
No answer	1.9 (8)
Perceived severity: Evaluation of anemia	*n*=418
Problem	77.0 (322)
No problem	1.0 (4)
Do not know	21.3 (89)
No answer	0.7 (3)
Attitudes towards iron-rich foods	
Perceived benefits: Evaluation of preparation of iron-rich foods	*n*=443
Good	71.6 (317)
Bad	2.7 (12)
Do not know	24.6 (109)
No answer	1.1 (5)
Perceived barriers: Difficulty of preparing iron-rich foods	*n*=425
Difficult	14.6 (62)
Neither difficult nor easy	31.1 (132)
Easy	9.2 (39)
Do not know	42.1 (179)
No answer	3.1 (13)
Self-confidence: Confidence in preparing iron-rich foods	*n*=439
Not confident	16.9 (74)
Ok / so-so	15.5 (68)
Confident	19.4 (85)
Do not know	40.5 (178)
No answer	7.7 (34)
Attitudes towards vitamin A deficiency	
Perceived susceptibility: Possibility of being vitamin A deficient	*n*=435
Possible	17.9 (78)
Not possible	29.4 (128)
Do not know	50.6 (220)
No answer	2.1 (9)
Perceived severity: Evaluation of vitamin A deficiency	*n*=438
Problem	69.4 (304)
No problem	2.5 (11)
Do not know	26.9 (118)
No answer	1.1 (5)
Attitudes towards vitamin A-rich foods	
Perceived benefits: Evaluation of preparation of vitamin A-rich foods	*n*=442
Good	71.9 (318)
Bad	1.8 (8)
Do not know	24.9 (110)
No answer	1.4 (6)

Perceived barriers: Difficulty of preparing vitamin A-rich foods	*n*=441
Difficult	17.0 (75)
Neither difficult nor easy	26.3 (116)
Easy	7.9 (35)
Do not know	44.0 (194)
No answer	4.8 (21)
Self-confidence: Confidence in preparing vitamin A-rich foods	*n*=442
Not confident	16.7 (74)
Ok / so-so	15.4 (68)
Confident	19.7 (87)
Do not know	42.3 (187)
No answer	5.9 (26)

[a] Categorical variables are expressed as % (n).

[b] Lack of corresponding sum of frequencies with total sample size is due to missing data; total frequencies per variable are given.

Table S6: Ability of justification for nutrition-related attitudes of lactating mothers

Variables [a]	Not possible, no problem, bad, difficult, not confident	Neither-nor, ok / so-so	Possible, problem, good, easy, confident	Total [b] *N*=444	*P*-value [c]	Effect size
Attitudes towards malnutrition						
Perceived susceptibility: Possibility of being malnourished	*n*=195		*n*=105	*n*=300	**<0.001†**	**0.280**
Can state a reason	62.1 (121)		86.7 (91)	70.7 (212)		
Do not know	37.4 (73)		11.4 (12)	28.3 (85)		
No answer	0.5 (1)		1.9 (2)	1.0 (3)		
Perceived severity: Evaluation of malnutrition	*n*=7		*n*=374	*n*=381	0.172†	0.075
Can state a reason	71.4 (5)		85.0 (318)	84.8 (323)		
Do not know	14.3 (1)		11.2 (42)	11.3 (43)		
No answer	14.3 (1)		3.7 (14)	3.9 (15)		
Attitudes towards anemia and iron-rich foods						
Perceived susceptibility: Possibility of being anemic	*n*=131		*n*=101	*n*=232	**<0.001**	**0.324**
Can state a reason	34.4 (45)		49.5 (50)	40.9 (95)		
Do not know	61.8 (81)		32.7 (33)	49.1 (114)		
No answer	3.8 (5)		17.8 (18)	9.9 (23)		
Perceived severity: Evaluation of anemia	*n*=1		*n*=320	*n*=321	0.346†	0.096
Can state a reason	0.0 (0)		65.6 (210)	65.4 (210)		
Do not know	100.0 (1)		25.0 (80)	25.2 (81)		
No answer	0.0 (0)		9.4 (30)	9.3 (30)		
Perceived benefits: Evaluation of preparation of iron-rich foods	*n*=12		*n*=314	*n*=326	0.875†	0.052
Can state a reason	50.0 (6)		54.1 (170)	54.0 (176)		
Do not know	50.0 (6)		40.8 (128)	41.1 (134)		
No answer	0.0 (0)		5.1 (16)	4.9 (16)		
Perceived barriers: Difficulty of preparing iron-rich foods	*n*=62	*n*=130	*n*=39	*n*=231	**<0.001†**	**0.303**
Can state a reason	29.0 (18)	45.4 (59)	87.2 (34)	48.1 (111)		
Do not know	45.2 (28)	46.2 (60)	12.8 (5)	40.3 (93)		
No answer	25.8 (16)	8.5 (11)	0.0 (0)	11.7 (27)		
Self-confidence: Confidence in preparing iron-rich foods	*n*=73	*n*=64	*n*=80	*n*=217	**<0.001**	**0.331**
Can state a reason	31.5 (23)	15.6 (10)	62.5 (50)	38.2 (83)		
Do not know	42.5 (31)	68.8 (44)	36.3 (29)	47.9 (104)		
No answer	26.0 (19)	15.6 (10)	1.3 (1)	13.8 (30)		
Attitudes towards vitamin A deficiency and vitamin A-rich foods						
Perceived susceptibility: Possibility of being vitamin A deficient	*n*=123		*n*=76	*n*=199	**<0.001†**	**0.408**
Can state a reason	29.3 (36)		68.4 (52)	44.2 (88)		
Do not know	68.3 (84)		26.3 (20)	52.3 (104)		
No answer	2.4 (3)		5.3 (4)	3.5 (7)		
Perceived severity: Evaluation of vitamin A deficiency	*n*=4		*n*=300	*n*=304	1.000†	0.031
Can state a reason	75.0 (3)		66.3 (199)	66.4 (202)		
Do not know	25.0 (1)		28.0 (84)	28.0 (85)		
No answer	0.0 (0)		5.7 (17)	5.6 (17)		

Perceived benefits: Evaluation of preparation of vitamin A-rich foods	*n*=7		*n*=307	*n*=314	0.349†	0.072
Can state a reason	57.1 (4)		64.8 (199)	64.6 (203)		
Do not know	28.6 (2)		30.9 (95)	30.9 (97)		
No answer	14.3 (1)		4.2 (13)	4.5 (14)		
Perceived barriers: Difficulty of preparing vitamin A-rich foods	*n*=74	*n*=112	*n*=32	*n*=218	**0.004†**	**0.190**
Can state a reason	55.4 (41)	41.1 (46)	75.0 (24)	50.9 (111)		
Do not know	31.1 (23)	50.0 (56)	21.9 (7)	39.4 (86)		
No answer	13.5 (10)	8.9 (10)	3.1 (1)	9.6 (21)		
Self-confidence: Confidence in preparing vitamin A-rich foods	*n*=74	*n*=63	*n*=80	*n*=217	**<0.001**	**0.327**
Can state a reason	21.6 (16)	19.0 (12)	60.0 (48)	35.0 (76)		
Do not know	56.8 (42)	73.0 (46)	38.8 (31)	54.8 (119)		
No answer	21.6 (16)	7.9 (5)	1.3 (1)	10.1 (22)		

[a] Categorical variables are expressed as % (n).

[b] Lack of corresponding sum of frequencies with total sample size is due to missing data; total frequencies per variable are given.

[c] Significantly different at p-value <0.05 (in bold); *P*-value was derived using chi-square analysis for categorical variables.

† Fisher's exact test.

Table S7: Nutrition-related attitudes and associated reasons of lactating mothers

**Variables** [a]	**Total** [b] *N*=444
Attitudes towards malnutrition	
Perceived susceptibility: Possibility of being malnourished	*n*=424
Possible	24.8 (105)
Not possible	46.9 (199)
Do not know	26.4 (112)
No answer	1.9 (8)
Reasons for malnutrition being possible[c]	*n*=105
Poor eating behavior (incl lack of food, lack of means, difficult life)	18.1 (19)
If poor eating behavior (incl lack of means)	53.3 (56)
Disease / bad for health / bad	4.8 (5)
If intestinal worms / no deworming	3.8 (4)
Exists, can happen to everyone	2.9 (3)
Being ill	1.9 (2)
If ill	2.9 (3)
If pregnant while having small baby	1.0 (1)
World changes	1.0 (1)
But I don't like (do not want/like)	1.0 (1)
Do not know	11.4 (12)
No answer	1.9 (2)
Reasons for malnutrition not being possible[c]	*n*=195
Good eating behavior (incl financial means)	31.3 (61)
If good eating behavior (incl financial means)	1.5 (3)
Shameful (disease)	8.7 (17)
Disease / bad for health / bad	8.7 (17)
Do not want / like	4.6 (9)
Being adult	3.1 (6)
Being healthy, no predisposition (incl knowledge)	1.0 (2)
Not existent in the family	1.0 (2)
Prevention, coping practices	1.0 (2)
Cultivation	1.0 (2)
Consulting medical staff	0.5 (1)
God	0.5 (1)
Do not know	37.4 (73)
No answer	0.5 (1)
Perceived severity: Evaluation of malnutrition	*n*=441
Problem	85.5 (377)
No problem	2.5 (11)
Do not know	11.6 (51)
No answer	0.5 (2)

Reasons for malnutrition being a problem[c]	*n*=374
Death / Disease leading to death	25.7 (96)
Disease (severe, dangerous)	25.7 (96)
Shameful (disease)	15.8 (59)
Change/destruction of body/skin(/hair)	9.4 (35)
Bad / bad for health	4.8 (18)
Weakness	2.4 (9)
Lack in the body / of food / of nutrient	2.4 (9)
Unwanted	1.1 (4)
Disease of the poor	1.1 (4)
Impaired (child) growth	0.8 (3)
Hardly heals	0.5 (2)
Bad for life	0.5 (2)
Lack of means	0.5 (2)
Disease of ignorants	0.3 (1)
Intestinal worms	0.3 (1)
Emotional imbalance	0.3 (1)
Great problem	0.3 (1)
Do not know	11.2 (42)
No answer	3.7 (14)
Reasons for malnutrition not being a problem[c]	*n*=7
Disease	42.9 (3)
Unwanted	14.3 (1)
Existence of nutritional center	14.3 (1)
Do not know	14.3 (1)
No answer	14.3 (1)
Attitudes towards anemia	
Perceived susceptibility: Possibility of being anemic	*n*=420
Possible	24.3 (102)
Not possible	32.1 (135)
Do not know	41.7 (175)
No answer	1.9 (8)
Reasons for anemia being possible[c]	*n*=101
Poor eating behavior (incl lack of means)	3.0 (3)
If poor eating behavior (incl lack of means)	22.8 (23)
Being ill	2.0 (2)
If ill	11.9 (12)
Disease / bad for health / bad	5.0 (5)
If blood loss/abortion	2.0 (2)
Exists, can happen to everyone	2.0 (2)
If neglecting	1.0 (1)
Do not know	32.7 (33)
No answer	17.8 (18)
Reasons for anemia not being possible[c]	*n*=131
Good eating behavior	18.3 (24)
Disease / bad for health / bad	5.3 (7)
Do not want / like	4.6 (6)
Knowledge	1.5 (2)
Not possible / not ill by anemia	1.5 (2)
Not existent in the family/community	1.5 (2)
Not being ill	0.8 (1)
Do not know anemia	0.8 (1)
God	0.8 (1)
Do not know	61.8 (81)
No answer	3.8 (5)
Perceived severity: Evaluation of anemia	*n*=418
Problem	77.0 (322)
No problem	1.0 (4)
Do not know	21.3 (89)
No answer	0.7 (3)

Reasons for anemia being a problem[c]	*n*=320
Disease (bad)	27.8 (89)
Death, fatal disease, diseases are fatal	25.3 (81)
Bad, bad for health/body	7.2 (23)
Weakness, fatigue, not able to work	2.8 (9)
Vertigo	1.9 (6)
Blood decrease	1.3 (4)
Malnutrition	0.6 (2)
Costly	0.3 (1)
Growth retardation	0.3 (1)
Pallor	0.3 (1)
Shameful	0.3 (1)
Weight loss	0.3 (1)
Disease that transforms the eyes	0.3 (1)
Do not know	25.0 (80)
No answer	9.4 (30)
Reasons for anemia not being a problem[c]	*n*=1
Do not know	100.0 (1)
Attitudes towards iron-rich foods	
Perceived benefits: Evaluation of preparation of iron-rich foods	*n*=443
Good	71.6 (317)
Bad	2.7 (12)
Do not know	24.6 (109)
No answer	1.1 (5)
Reasons for good value of iron-rich foods[c]	*n*=314
Good for health	29.9 (94)
Increasing blood	8.0 (25)
Increasing iron	7.0 (22)
Increasing strength/energy	1.9 (6)
Construction, protection	1.9 (6)
Preventing disease, death	1.6 (5)
Rich in/increasing nutrients/vitamins	1.6 (5)
Preventing anemia	1.0 (3)
Help, good life	1.0 (3)
Satisfying	0.6 (2)
Like	0.3 (1)
For cooking	0.3 (1)
Good	0.3 (1)
Preventing malnutrition	0.3 (1)
Available	0.3 (1)
Tasty	0.3 (1)
Treat the problem	0.3 (1)
Other	0.3 (1)
Do not know	40.8 (128)
No answer	5.1 (16)
Reasons for no/bad value of iron-ich foods[c]	*n*=12
Excess is not beneficial neither	50.0 (6)
Do not know	50.0 (6)
Perceived barriers: Difficulty of preparing iron-rich foods	*n*=425
Difficult	14.6 (62)
Neither difficult nor easy	31.1 (132)
Easy	9.2 (39)
Do not know	42.1 (179)
No answer	3.1 (13)
Reasons for preparation of iron-rich foods being difficult[c]	*n*=62
Lack of financial means	14.5 (9)
Lack of knowledge (about iron-rich foods)	11.3 (7)
Time-consuming	1.6 (1)
Gives strength	1.6 (1)
Do not know	45.2 (28)
No answer	25.8 (16)

Reasons for preparation of iron-rich foods being neither difficult nor easy[c]	*n*=130
According to financial means / if means	25.4 (33)
According to availability / if available	6.2 (8)
Depending on knowledge / if knowledge (about iron-rich foods)	4.6 (6)
Lack of knowledge (about iron-rich foods)	3.8 (5)
Availability of iron-rich foods	3.1 (4)
Considering it + coping to have them	1.5 (2)
Bad life	0.8 (1)
Preparing everything	0.8 (1)
Standard meal	0.8 (1)
Good for health	0.8 (1)
Do not know	46.2 (60)
No answer	8.5 (11)
Reasons for preparation of iron-rich foods being easy[c]	*n*=39
Availability of iron-rich foods	46.2 (18)
Knows/used to preparing, easy to prepare	12.8 (5)
Availability of ember/coal	7.7 (3)
Depending on availability of necessary / if necessary available	7.7 (3)
Affordability of iron-rich foods	5.1 (2)
According to financial means / if means	5.1 (2)
Not demanding	2.6 (1)
Does not have defense	2.6 (1)
Good for health	2.6 (1)
Do not know	12.8 (5)
Self-confidence: Confidence in preparing iron-rich foods	*n*=439
Not confident	16.9 (74)
Ok / so-so	15.5 (68)
Confident	19.4 (85)
Do not know	40.5 (178)
No answer	7.7 (34)
Reasons for being not confident in preparation of iron-rich foods[c]	*n*=73
Lack of financial means	15.1 (11)
Lack of knowledge (about iron-rich foods)	13.7 (10)
Not being a professional	1.4 (1)
No dietary diversity	1.4 (1)
Do not know	42.5 (31)
No answer	26.0 (19)
Reasons for being ok with preparation of iron-rich foods[c]	*n*=64
Good for health	4.7 (3)
Availability of iron-rich foods	3.1 (2)
According to/depending on financial means / if means	3.1 (2)
Lack of knowledge (about iron-rich foods)	1.6 (1)
Good eating behavior (well, iron-rich foods)	1.6 (1)
Like	1.6 (1)
Do not know	68.8 (44)
No answer	15.6 (10)
Reasons for being confident in preparation of iron-rich foods[c]	*n*=80
Good for health	28.7 (23)
Preparing well / iron-rich foods	10.0 (8)
Food / good food / good	8.8 (7)
Good eating behavior (well, iron-rich foods)	5.0 (4)
Like	5.0 (4)
Normal, as a woman	3.8 (3)
Availability of iron-rich foods	2.5 (2)
Working well	1.3 (1)
Do not know	36.6 (29)
No answer	1.3 (1)

Attitudes towards vitamin A deficiency	
Perceived susceptibility: Possibility of being vitamin A deficient	*n*=435
Possible	17.9 (78)
Not possible	29.4 (128)
Do not know	50.6 (220)
No answer	2.1 (9)
Reasons for vitamin A deficiency being possible[c]	*n*=76
Poor eating behavior (incl lack of means)	18.4 (14)
If poor eating behavior (incl lack of means)	35.5 (27)
Being ill	2.6 (2)
If ill	3.9 (3)
No knowledge about vitamin A-rich foods	2.6 (2)
If no knowledge about vitamin A-rich foods	3.9 (3)
If blood decrease	1.3 (1)
Can happen	1.3 (1)
Vertigo	1.3 (1)
Do not know	26.3 (20)
No answer	5.3 (4)
Reasons for vitamin A deficiency not being possible[c]	*n*=123
Good eating behavior	18.7 (23)
Disease / bad for health / bad	3.3 (4)
Being healthy / not ill	2.4 (3)
If financial means	1.6 (2)
Knowledge about vitamin A-rich foods	1.6 (2)
If knowledge about vitamin A-rich foods	0.8 (1)
Do not want	0.8 (1)
Do not know vitamin A	0.8 (1)
Avoid bad situation	0.8 (1)
Do not know	68.3 (84)
No answer	2.4 (3)
Perceived severity: Evaluation of vitamin A deficiency	*n*=438
Problem	69.4 (304)
No problem	2.5 (11)
Do not know	26.9 (118)
No answer	1.1 (5)
Reasons for vitamin A deficiency being a problem[c]	*n*=300
Disease	27.7 (83)
Death, fatal disease, diseases are fatal	11.0 (33)
Weakness, fatigue	9.7 (29)
Vision loss, eye problems, blindness	5.7 (17)
Bad, bad for health/body	5.0 (15)
Causes disease, susceptible for disease, impaired immune system	5.0 (15)
Malnutrition, kwashiorkor, deficiency, eating poorly	2.0 (6)
Weight loss, no weight gain	1.0 (3)
Importance of vitamin A	0.7 (2)
Already affected	0.3 (1)
Expensive care	0.3 (1)
Lack of care	0.3 (1)
Vaccination only for dangerous diseases	0.3 (1)
Blood decrease	0.3 (1)
Do not know	28.0 (84)
No answer	5.7 (17)
Reasons for vitamin A deficiency not being a problem[c]	*n*=4
Vitamin A in spring water	25.0 (1)
Vitamin A in meals	25.0 (1)
Preparation possible if knowledge	25.0 (1)
Do not know	25.0 (1)

Attitudes towards vitamin A-rich foods	
Perceived benefits: Evaluation of preparation of vitamin A-rich foods	*n*=442
Good	71.9 (318)
Bad	1.8 (8)
Do not know	24.9 (110)
No answer	1.4 (6)
Reasons for good value of vitamin A-rich foods[c]	*n*=307
Good, good for health/body	33.6 (103)
Providing/increasing vitamin A	12.7 (39)
Increase of energy/strength	5.5 (17)
Providing/increasing nutrients/vitamins	5.5 (17)
Protection (against diseases), improving immune system	3.9 (12)
Good for vision, protection of eyes, avoiding vision loss	1.6 (5)
Good for growth	1.3 (4)
Construction	1.0 (3)
Increase of blood	0.7 (2)
Brings breast milk	0.3 (1)
Easy	0.3 (1)
Do not know	30.9 (95)
No answer	4.2 (13)
Reasons for no/bad value of vitamin A-rich foods[c]	*n*=7
Excess is not beneficial neither	57.1 (4)
Do not know	28.6 (2)
No answer	14.3 (1)
Perceived barriers: Difficulty of preparing vitamin A-rich foods	*n*=441
Difficult	17.0 (75)
Neither difficult nor easy	26.3 (116)
Easy	7.9 (35)
Do not know	44.0 (194)
No answer	4.8 (21)
Reasons for preparation of vitamin A-rich foods being difficult[c]	*n*=74
Lack of financial means	31.1 (23)
Lack of knowledge (about vitamin A/vitamin A-rich foods)	23.0 (17)
Not available	1.4 (1)
According to/depending on/if financial means	1.4 (1)
Do not know	31.1 (23)
No answer	13.5 (10)
Reasons for preparation of vitamin A-rich foods being neither difficult nor easy[c]	*n*=112
According to/depending on/if financial means	24.1 (27)
According to/depending on/if availability	6.9 (7)
Lack of financial means	2.7 (3)
Lack of knowledge (about vitamin A/vitamin A-rich foods)	2.7 (3)
Depending on if knowledge (about vitamin A-rich foods)	1.8 (2)
Contained in usual food	1.8 (2)
Available	0.9 (1)
Knows/used to preparing, easy to prepare	0.9 (1)
Financial means	0.9 (1)
Coping for good diet	0.9 (1)
Do not know	50.0 (56)
No answer	8.9 (10)

Reasons for preparation of vitamin A-rich foods being easy[c]	*n*=32
Available	28.1 (9)
Affordable	15.6 (5)
Knows/used to preparing, easy to prepare	15.6 (5)
According to/depending on/if availability	9.4 (3)
According to/depending on/if financial means	3.1 (1)
Depending on/if knowledge (about vitamin A-rich foods)	3.1 (1)
Good meal	3.1 (1)
Do not know	21.9 (7)
No answer	3.1 (0)
Self-confidence: Confidence in preparing vitamin A-rich foods	*n*=442
Not confident	16.7 (74)
Ok / so-so	15.4 (68)
Confident	19.7 (87)
Do not know	42.3 (187)
No answer	5.9 (26)
Reasons for being not confident in preparation of vitamin A-rich foods[c]	*n*=74
Lack of financial means	12.2 (9)
Lack of knowledge (about VA-rich foods)	9.5 (7)
Not available/no means	1.4 (1)
Do not know	56.8 (42)
No answer	21.6 (16)
Reasons for being ok with preparation of vitamin A-rich foods[c]	*n*=63
According to/depending on/if financial means	4.8 (3)
Good, good for health	3.2 (2)
Good meal, increasing nutrients/vitamins/vitamin A/ energy/strength/blood, satisfying	1.6 (1)
Financial means	1.6 (1)
Like	1.6 (1)
Knowledge (about vitamin A-rich/important foods)	1.6 (1)
Lack of knowledge (about vitamin A-rich foods)	1.6 (1)
Depending on knowledge	1.6 (1)
Lack of financial means	1.6 (1)
Seems to increase weight	1.6 (1)
Do not know	73.0 (46)
No answer	7.9 (5)
Reasons for being confident in preparation of vitamin A-rich foods[c]	*n*=80
Good, good for health	20.0 (16)
Good meal, increasing nutrients/vitamins/vitamin A/ energy/strength/blood, satisfying	17.5 (14)
Preparing well, used of preparing	8.8 (7)
Like	5.0 (4)
Available	3.8 (3)
According to/depending on/if financial means	2.5 (2)
Good eating behavior (well)	1.3 (1)
If available	1.3 (1)
Increasing	1.3 (1)
Knowledge (about vitamin A-rich/important foods)	1.3 (1)
Do not know	38.8 (31)
No answer	1.3 (1)

[a] Categorical variables are expressed as % (n).

[b] Lack of corresponding sum of frequencies with total sample size is due to missing data; total frequencies per variable are given.

[c] Multiple response question.

Table S8: Food group consumption of lactating mothers

Variables [a]	Total [b] *N*=411
Starchy staples	99.8 (410)
Grains	71.8 (295)
White roots and tubers, plantains	85.2 (350)
Pulses	29.7 (122)
Soy	10.7 (44)
Beans, lentils	20.7 (85)
Nuts and seeds	14.4 (59)
Milk and milk products	6.3 (26)
Fresh milk	0.2 (1)
Powdered milk	5.4 (22)
Cheese, yogurt, dairy products	0.7 (3)
Meat, poultry, fish	47.4 (195)
Meat	8.0 (33)
Poultry	0.5 (2)
Organs	0.2 (1)
Fish and seafood	41.4 (170)
Eggs	0.2 (1)
Dark green leafy vegetables	64.5 (265)
Other vitamin A-rich fruits and vegetables	1.0 (4)
Other vitamin A-rich fruits	1.0 (4)
Other vitamin A-rich vegetables	0.0 (0)
Other vegetables	60.8 (250)
Other fruits	11.7 (48)
Oils and fats	77.9 (320)
Vegetable oil, other fats	29.4 (121)
Palm oil	57.7 (237)
Unhealthy foods	26.0 (107)
Fried and salty foods	2.4 (10)
Sweet foods	10.2 (42)
Sweet beverages	16.3 (67)
Alcoholic beverages	0.7 (3)
Condiments and seasonings	90.0 (370)
Other beverages and foods	0.5 (2)

[a] Categorical variables are expressed as % (n).

[b] Lack of corresponding sum of frequencies with total sample size is due to missing data and mothers not eating at the reported day.

Table S9: K-, P-Scores, DDS, and Hb concentration of lactating mothers and infants by maternal educational level

Variables [a]	Low educational level	Elementary educational level	Secondary educational level	Total [b]	*P*-value [c]
	n=71	*n*=140	*n*=230	*n*=441	
K-Score *3 food group model*	*n*=69<br>0.42 ± 0.34<br>0.25<br>(0.25, 0.75)	*n*=137<br>0.33 ± 0.35<br>0.25<br>(0.00, 0.50)	*n*=229<br>0.36 ± 0.34<br>0.25<br>(0.00, 0.75)	*n*=435<br>0.36 ± 0.35<br>0.25<br>(0.00, 0.75)	0.146
K-Score *malnutrition*	*n*=70<br>0.79 ± 0.32<br>1.00<br>(0.75, 1.00)	*n*=138<br>0.74 ±0.34<br>1.00<br>(0.50, 1.00)	*n*=230<br>0.77 ± 0.33<br>1.00<br>(0.50, 0.75)	*n*=438<br>0.76 ± 0.33<br>1.00<br>(0.50, 1.00)	0.318
K-Score *iron + anemia*	*n*=69<br>0.27 ± 0.32<br>0.14<br>(0.00, 0.57)	*n*=134<br>0.24 ± 0.30<br>0.00<br>(0.00, 0.45)	*n*=222<br>0.23 ± 0.31<br>0.00<br>(0.00, 0.50)	*n*=425<br>0.24 ± 0.31<br>0.00<br>(0.00, 0.50)	0.467
K-Score *vitamin A + VAD*	*n*=69<br>0.39 ± 0.40<br>0.33[i]<br>(0.00, 0.75)	*n*=138<br>0.41 ± 0.41<br>0.33[i]<br>(0.00, 0.75)	*n*=225<br>0.25 ± 0.38<br>0.00[ii]<br>(0.00, 0.75)	*n*=432<br>0.33 ± 0.40<br>0.00<br>(0.00, 0.75)	**<0.001**
K-Score *total*	*n*=70<br>0.45 ± 0.25<br>0.44<br>(0.24, 0.64)	*n*=136<br>0.42 ± 0.23<br>0.39<br>(0.24, 0.56)	*n*=230<br>0.39 ± 0.23<br>0.35<br>(0.24, 0.56)	*n*=436<br>0.41 ± 0.24<br>0.39<br>(0.24, 0.56)	0.139
P-Score *iron*	*n*=70<br>0.43 ±0.15<br>0.50[i, ii]<br>(0.33, 0.50)	*n*=135<br>0.40 ± 0.15<br>0.33[i]<br>(0.33, 0.50)	*n*=218<br>0.45 ± 0.13<br>0.50[ii]<br>(0.33, 0.50)	*n*=423<br>0.43 ± 0.14<br>0.50<br>(0.33, 0.50)	**0.009**
Dietary Diversity Score	*n*=64<br>3.2 ± 1.1<br>3.0<br>(2.0, 4.0)	*n*=134<br>3.3 ± 1.0<br>3.0<br>(3.0, 4.0)	*n*=211<br>3.5 ± 1.2<br>3.0<br>(3.0, 4.0)	*n*=409<br>3.4 ± 1.1<br>3.0<br>(3.0, 4.0)	0.201
Maternal Hb	*n*=69<br>12.4 ± 1.8<br>12.7<br>(11.3, 13.7)	*n*=139<br>12.5 ± 1.6<br>12.8<br>(12.0, 13.6)	*n*=225<br>12.5 ± 1.4<br>12.7<br>(11.7, 13.5)	*n*=433<br>12.5 ± 1.5<br>12.7<br>(11.7, 13.5)	0.883
Infantile Hb	*n*=70<br>10.2 ± 1.5<br>10.2<br>(9.3, 11.1)	*n*=140<br>10.3 ± 1.6<br>10.3<br>(9.6, 11.1)	*n*=226<br>10.1 ± 1.4<br>9.9<br>(9.2, 10.9)	*n*=436<br>10.2 ± 1.5<br>10.1<br>(9.3, 11.0)	0.136

[a] Metric variables are expressed as mean ± SD and median (IQR).

[b] Lack of corresponding sum of frequencies with total sample size is due to missing data; total frequencies per variable are given.

[c] Significantly different at *P*-value <0.05 (in bold); *P*-value was derived using Kruskal-Wallis test for metric variables.

[i, ii] Groups with differing characters differ significantly.

VAD: vitamin A deficiency.
